# Performance of Large Language Models for Oncology Nursing Decision Support: Cross-Sectional Study

**DOI:** 10.2196/97802

**Published:** 2026-07-24

**Authors:** Qiongyu Zhou, Yan Jia, Haiqin Hu, Danjiao Huang, Xuefeng Chen, Yirong Xia, Wanying Wu

**Affiliations:** 1 School of Nursing, Zhejiang Chinese Medical University, Hangzhou, Zhejiang, China; 2 Hangzhou Institute of Medicine, Chinese Academy of Sciences, Zhejiang Cancer Hospital, Hangzhou, Zhejiang, 310022, China, 86 13857137426

**Keywords:** large language models, artificial intelligence, AI, natural language processing, nursing, oncology nursing, clinical decision support systems

## Abstract

**Background:**

Large language models (LLMs) are increasingly used in health care, with emerging applications in clinical decision support and nursing education. However, evidence on their performance in nursing contexts, particularly in oncology nursing, remains limited. Given the complexity and high-risk nature of oncology care, it is important to evaluate the performance and clinical relevance of LLM-generated responses in oncology nursing contexts.

**Objective:**

This study aimed to compare the performance of LLMs in oncology nursing decision support tasks using standardized examination questions and case-based clinical scenarios and explore LLMs’ potential applicability and current limitations in oncology nursing practice.

**Methods:**

A total of 33 case-based questions derived from 10 oncology nursing clinical scenarios in a nationally used training manual, along with standardized examination-oriented questions from a commercially published preparation book for the Chinese Nursing (Intermediate) Qualification Examination, were used to evaluate the performance of 5 LLMs (DeepSeek, Qwen, Spark-Desk, WiseDiag, and ChatGPT). All models generated responses using a standardized prompt. Two oncology nurses with more than 5 years of clinical experience independently rated the case-based responses using 3 evaluation dimensions: correctness, clarity, and conciseness. Interrater reliability was assessed using the quadratic weighted Cohen κ, intraclass correlation coefficient, and Spearman rank correlation coefficient. Differences among models were analyzed using the Kruskal-Wallis test with the Dunn post hoc test. In addition, examination performance was evaluated based on total score, accuracy rate, and completion efficiency.

**Results:**

Interrater reliability analyses indicated moderate agreement between evaluators. The median correctness, clarity, and conciseness scores were as follows: 11.50 (IQR 10.50-12.00) for DeepSeek, 11.00 (IQR 10.50-12.00) for Qwen, 10.50 (IQR 9.50-11.50) for Spark-Desk, 10.00 (IQR 9.50-11.50) for WiseDiag, and 10.00 (IQR 9.00-11.50) for ChatGPT. The Kruskal-Wallis test indicated statistically significant differences among models (*H*=11.416; *P*<.05), with post hoc analysis showing a significant difference only between DeepSeek and ChatGPT (*P*<.05). In examination-based tasks, all models achieved passing performance, with accuracy rates ranging from 77% (77/100) to 93% (93/100). In terms of response completion, DeepSeek and ChatGPT completed all tasks in a single interaction, whereas other models required multiple interactions due to output interruptions.

**Conclusions:**

LLMs showed relatively strong performance on structured knowledge and examination-based tasks but remained limited in complex oncology nursing scenarios requiring individualized assessment and dynamic clinical judgment. Their potential use may be most relevant to information retrieval, knowledge organization, and patient education. Because the correctness, clarity, and conciseness rubric showed only moderate interrater reliability, the case-based comparisons should be interpreted as preliminary signals rather than definitive evidence of between-model differences. LLM outputs should therefore be used as supportive information and interpreted alongside professional clinical judgment.

## Introduction

Oncology nursing is characterized by complex symptom management, rapidly evolving patient conditions, and high-stakes clinical decision-making [1,2]. Nurses are required to continuously integrate multidimensional information, including treatment regimens, adverse effects, and patient-specific factors, often under conditions of uncertainty and time pressure. As cancer care becomes increasingly intensive and individualized, the cognitive demands placed on oncology nurses continue to grow, highlighting the need for efficient and effective clinical decision support tools [3].

In this context, AI has rapidly advanced and is increasingly integrated into health care practice [4-7]. Among these developments, large language models (LLMs) have attracted particular attention due to their capacity to process natural language, synthesize dispersed information, and generate contextually relevant responses. Unlike traditional retrieval-based systems, LLMs enable interactive, dialogue-based support, allowing clinicians to access synthesized knowledge without navigating complex search processes [8]. These features suggest potential utility in supporting clinical tasks that involve high information density and time constraints.

A growing body of research has explored the application of LLMs in medical contexts. Studies have demonstrated that models such as ChatGPT can achieve competitive performance in standardized medical licensing examinations, indicating a solid foundation in medical knowledge representation [6,9]. Other work has examined LLMs’ roles in patient education, clinical communication, and preliminary decision support, further supporting their potential utility in health care settings [10,11]. However, existing evidence has largely focused on general medical scenarios, with relatively limited attention to complex nursing contexts, particularly oncology nursing. Compared with general medical tasks, oncology nursing involves more dynamic symptom trajectories, stricter safety thresholds, and a greater reliance on continuous assessment and timely intervention [1,2,12]. These features place higher demands on both the accuracy and contextual adaptability of decision support tools.

In addition, most existing studies have focused on single-model evaluations, typically centered on ChatGPT, whereas the broader landscape of LLMs remains underexplored. In recent years, the development of LLMs has accelerated worldwide, accompanied by the rapid emergence of models developed in different linguistic and technological environments. These models differ not only in training data composition but also in architectural design and optimization strategies, which may influence their performance in clinically relevant tasks. This is particularly important because the performance of LLMs is closely associated with the linguistic distribution and domain characteristics of their training corpora. Previous studies have shown that models trained predominantly on English-language data may exhibit performance variability when applied to non–English-language clinical contexts, where differences in language use, medical knowledge representation, and clinical practice patterns exist [13]. Moreover, comparative research has demonstrated measurable differences between internationally developed models and those trained on Chinese-language corpora in medical tasks, suggesting that these variations are not solely due to language barriers but are also influenced by disparities in training data and model design [14,15]. Therefore, restricting evaluation to a single model provides only a limited understanding of current model capabilities. A comparative assessment across multiple models is necessary to capture the heterogeneity of performance and better reflect the range of technologies available in real-world clinical settings.

Beyond accuracy, other performance characteristics are also critical for clinical use. In nursing practice, decision support outputs must be readily interpretable, with sufficient clarity and conciseness to support timely decision-making while minimizing additional cognitive burden [16]. In addition, the completeness of model-generated responses may influence usability in complex or information-dense clinical scenarios, particularly when tasks require extensive information processing or lengthy outputs. These factors may affect the practicality of integrating LLM outputs into routine clinical workflows [17]. Standardized nursing examination questions, particularly case-based items, encapsulate key elements of clinical decision-making, including symptom progression, risk prioritization, and the timing of interventions [14]. As such, they provide a structured and clinically relevant reference for evaluating the performance of LLMs in oncology nursing contexts. Therefore, this study aimed to compare the performance of multiple LLMs in oncology nursing decision support tasks using standardized examination questions and case-based clinical scenarios and explore LLMs’ potential applicability and current limitations in oncology nursing practice.

## Methods

### Study Design

This study used a comparative cross-sectional design to evaluate the performance of multiple LLMs in oncology nursing decision support tasks. The evaluation was conducted as an observational in silico assessment focusing on the models’ ability to generate nursing-relevant responses to standardized clinical scenarios. No model retraining, fine-tuning, or parameter modification was performed. All models were assessed in their publicly accessible form, reflecting their real-world use by frontline nursing professionals. This study was reported with reference to relevant items from the STROBE (Strengthening the Reporting of Observational Studies in Epidemiology) statement to improve transparency in reporting study design, data sources, data collection procedures, outcome measures, and statistical analyses.

### Study Units and Setting

The primary study units were the responses generated by the evaluated LLMs. These responses were elicited using 2 types of question sets: case-based questions and standardized examination questions. The case-based questions were derived from a nationally used oncology nursing training manual developed by the oncology training center of our institution and published in 2021. This manual is routinely used in the training and assessment of oncology nurses and contains structured clinical scenarios representing common and complex situations in practice. All case-based questions contained in the manual were extracted in full and used in this study to ensure comprehensive coverage of the source material. Therefore, no a priori sample size calculation was performed as this study was based on the complete set of available standardized questions from the selected sources rather than a sampled subset. These scenarios require integration of symptom assessment, complication recognition, and clinical decision-making, thereby closely reflecting real-world oncology nursing practice. The examination questions were selected from a commercially published examination preparation book for the Chinese Nursing (Intermediate) Qualification Examination published by Liaoning Science and Technology Press in 2025. The resource contains standardized examination-oriented questions covering core nursing knowledge and competencies. In the present study, these questions were used as a standardized examination-based benchmark to compare model performance across commonly tested nursing knowledge domains.

### Data Collection

Data were collected by evaluating the performance of multiple LLMs on case-based questions and standardized examination questions. Five widely used generative AI chatbots were selected for comparison: DeepSeek, Qwen (Alibaba Cloud), Spark-Desk (iFlytek), WiseDiag, and ChatGPT (OpenAI). The selection aimed to include both internationally established and rapidly evolving Chinese-language LLMs as well as systems with different training characteristics and optimization strategies to capture variability in model performance across diverse technical and linguistic backgrounds. Case-based evaluations were conducted between October 2025 and November 2025 through the official public web interfaces of the evaluated models. The standardized examination assessment was completed in December 2025. All models were evaluated using the publicly available versions accessible to users at the time of data collection. Exact model version identifiers were not consistently displayed or available through the public web interfaces of all evaluated platforms. No attempts were made to access archived model versions or developer-specific configurations. ChatGPT was evaluated using the free web-based version available to the general public. DeepSeek was evaluated through its official web interface using the Fast Mode setting. Qwen, Spark-Desk, and WiseDiag were accessed through their respective official web-based platforms using the default configurations available to public users during the study period. No API access, model switching, parameter adjustment, or prompt optimization was performed. Each model was presented with identical questions using a standardized prompt: “Please answer the following question. Provide only the final answer without explanation.” The same prompt was applied to both case-based questions and standardized examination questions across all evaluated models. All questions were administered in a single-turn format. Each case set was initiated in a new session to minimize potential contextual influence from previous interactions. No additional clarification or follow-up prompts were provided. Following the approach described by Gilson et al [18], each model was prompted once per question without repeated sampling. All responses were recorded verbatim for subsequent analysis.

### Measures

The performance of the LLMs was evaluated using both qualitative rating measures and objective examination-based indicators. For case-based questions, responses generated by each model were independently evaluated by 2 oncology nurses with more than 5 years of clinical experience. The raters were blinded to the identity of the models to minimize potential bias. Interrater reliability analyses were conducted using the initial independent ratings. The reported model performance scores were based on the mean ratings of the 2 raters. Response quality was assessed using 3 evaluation dimensions, namely, correctness, clarity, and conciseness. These dimensions have been frequently used in previous evaluations of LLM-generated responses in health care and medical education settings [19,20]. In the present study, they were used to assess the accuracy, comprehensibility, and efficiency of information delivery in oncology nursing scenarios. Correctness referred to the accuracy of the content, clarity reflected the comprehensibility and coherence of the response, and conciseness indicated the efficiency of information delivery. Each dimension was rated on a 4-point Likert scale. A score of 4 indicated a completely correct, clear, or concise response; a score of 3 indicated a mostly correct, clear, or concise response; a score of 2 indicated a partially correct, clear, or concise response; and a score of 1 indicated a completely incorrect, unclear, or nonconcise response. For examination-based tasks, objective indicators included total score and accuracy rate. Consistent with the passing standard of the Nursing (Intermediate) Qualification Examination, a score of 60% was considered the minimum competency threshold.

### Statistical Analysis

Interrater reliability was assessed using quadratic weighted Cohen κ for each of the 3 evaluation dimensions and a 2-way random-effects intraclass correlation coefficient (ICC) for the total correctness, clarity, and conciseness score. CIs for the weighted κ values and ICCs were estimated using bootstrap resampling of paired ratings. To facilitate comparison with the original analysis, the Spearman rank correlation coefficient was also calculated between the 2 raters’ total scores. Agreement and disagreement patterns were further summarized using the proportions of exact agreement, 1-point differences, and differences of 2 points or more. All tests were 2-tailed, and a *P* value below .05 was considered statistically significant. Prism (version 10.1.2; GraphPad Software) was used for additional analyses. The Shapiro-Wilk test indicated that the data were not normally distributed; therefore, results were summarized as medians with IQRs. Differences among the 5 LLMs were evaluated using the Kruskal-Wallis test (2-sided; α=.05). When a significant overall difference was observed, pairwise comparisons were conducted using the Dunn post hoc test, with *P* values adjusted using the false discovery rate method to control for multiple testing. Adjusted *P* values below .05 were considered statistically significant.

### Ethical Considerations

This study primarily involved the evaluation of outputs generated by LLMs using standardized oncology nursing questions and case-based clinical scenarios. The study protocol was submitted to the Ethics Committee of Zhejiang Cancer Hospital and was determined to be exempt from full ethics review (exemption record number 202606291151000205900). The exemption was granted because this study did not involve patients, clinical interventions, identifiable personal data, sensitive personal information, or clinical decision-making activities. Institutional permission to use the 2021 oncology nursing training manual for research and publication purposes was obtained from the oncology training center of our institution. The objective assessment component was based on standardized nursing examination materials that did not contain patient-related, identifiable, or sensitive information. The subjective evaluation involved professional assessment of model-generated outputs by experienced oncology nurses using a predefined evaluation framework. All participating nurses were informed of the study purpose and evaluation procedures, and informed consent was obtained prior to participation.

## Results

### Case-Based Evaluation

The weighted κ values were 0.466 for correctness, 0.521 for clarity, and 0.453 for conciseness. The ICC for the total correctness, clarity, and conciseness score was 0.554. Across 165 model-generated responses, identical total scores were assigned in 72 (43.6%) cases. Differences of 1 point and 2 points or more were observed in 28.5% (n=47) and 27.9% (n=46) of cases, respectively. Detailed interrater agreement and disagreement statistics are shown in Table 1. For comparison with the original analysis, the Spearman rank correlation coefficient between raters was 0.566 (*P*<.01). On the basis of 33 case-based questions derived from 10 clinical scenarios, the performance of the 5 LLMs is shown in Table 2. DeepSeek achieved the highest median score of 11.50 (IQR 10.50-12.00). Qwen and Spark-Desk obtained median scores of 11.00 (IQR 10.50-12.00) and 10.50 (IQR 9.50-11.50), respectively. WiseDiag and ChatGPT scored a median of 10.00 (IQR 9.50-11.50) and 10.00 (IQR 9.00-11.50), respectively. The Kruskal-Wallis test revealed a statistically significant difference in overall correctness, clarity, and conciseness scores among the 5 LLMs (*H*=11.416; *P=*.02). Post hoc analysis showed a significant difference only between DeepSeek and ChatGPT (raw *P*=.008; false discovery rate–adjusted *P*=.04). No significant differences were found among the remaining comparisons.

**Table 1.** Interrater reliability, agreement, and disagreement across the 3 evaluation dimensions (n=165 model-generated responses)^a^.

Dimension	Weighted κ (95% CI)	Exact agreement, n (%)	1-point difference, n (%)	≥2-point difference, n (%)
Correctness	0.466 (0.305‐0.607)	103 (62.4)	50 (30.3)	12 (7.3)
Clarity	0.521 (0.341‐0.676)	122 (73.9)	29 (17.6)	14 (8.5)
Conciseness	0.453 (0.270‐0.614)	123 (74.5)	25 (15.2)	17 (10.3)

a The 2-way random-effects intraclass correlation coefficient for absolute agreement on the total correctness, clarity, and conciseness score was 0.554.

**Table 2.** Scores of the 5 large language models^a^.

Model	Questions, n	Score (range 3-12), median (IQR)
DeepSeek	33	11.50 (10.50-12.00)^b^
Qwen	33	11.00 (10.50-12.00)
Spark-Desk	33	10.50 (9.50-11.50)
WiseDiag	33	10.00 (9.50-11.50)
ChatGPT	33	10.00 (9.00-11.50)^b^

a *H*=11.416; *P*=.02.

b The pairwise comparison between DeepSeek and ChatGPT was statistically significant (false discovery rate–adjusted P=.04).

### Standardized Examination Assessment

Performance on the standardized examination questions is shown in Table 3. DeepSeek achieved the highest examination score at 93 and an accuracy of 93% (93/100). ChatGPT ranked second, scoring 88 with an accuracy of 88% (88/100). WiseDiag and Spark-Desk followed, with scores of 85 and 84, corresponding to accuracies of 85% (85/100) and 84% (84/100), respectively. Qwen showed the lowest performance, scoring 77 with an accuracy of 77% (77/100). According to the Nursing (Intermediate) Qualification Examination, all 5 evaluated LLMs achieved a passing level. Regarding completion of the full question set, only DeepSeek and ChatGPT completed all 100 questions within a single session. Qwen and WiseDiag required 2 sessions to provide responses for all questions, whereas Spark-Desk required 5 sessions during data collection. These observations describe the number of sessions required to obtain complete responses under the study conditions.

**Table 3.** Performance of the 5 large language models on qualification examination questions.

Model	Correct answers (score) in internal medicine (n=34), n (%)^a^	Correct answers (score) in surgery (n=22), n (%)^b^	Correct answers (score) in gynecology (n=9), n (%)^c^	Correct answers (score) in pediatrics (n=16), n (%)^d^	Correct answers (score) in emergency and critical care (n=19), n (%)^e^	Overall (n=100)^f^	
						Sessions, n	Correct answers (score), n (%)
DeepSeek	30 (88.2)	21 (95.5)	9 (100)	14 (87.5)	19 (100)	1	93 (93)
Qwen	23 (67.6)	19 (86.4)	9 (100)	14 (87.5)	12 (63.2)	2	77 (77)
Spark-Desk	27 (79.4)	17 (77.3)	9 (100)	14 (87.5)	17 (89.5)	5	84 (84)
WiseDiag	28 (82.4)	19 (86.4)	8 (88.9)	13 (81.3)	17 (89.5)	2	85 (85)
ChatGPT	29 (85.3)	19 (86.4)	8 (88.9)	14 (87.5)	18 (94.7)	1	88 (88)

a Mean correct answers 27.4 (SD 2.7); mean accuracy 80.6% (SD 7.9%).

b Mean correct answers 19.0 (SD 1.4); mean accuracy 86.4% (SD 6.4%).

c Mean correct answers 8.6 (SD 0.5); mean accuracy 95.6% (SD 6.1%).

d Mean correct answers 13.8 (SD 0.4); mean accuracy 86.3% (SD 2.8%).

e Mean correct answers 16.6 (SD 2.7); mean accuracy 87.4% (SD 14.2%).

f Mean sessions 2.2 (SD 1.6); mean correct answers 85.4 (SD 5.9); mean accuracy 85.4% (SD 5.9%).

## Discussion

### Main Findings

The present study compared the performance of 5 widely used LLMs in oncology nursing decision support tasks using both case-based clinical scenarios and standardized examination questions. Overall, all evaluated models achieved passing scores on the examination assessment and obtained median correctness, clarity, and conciseness scores ranging from 10.00 to 11.50 in the case-based evaluation. DeepSeek achieved the highest score in the case-based assessment, whereas ChatGPT ranked second in the examination-based evaluation. These findings suggest that model performance may vary according to the type of task being evaluated. Examination performance also varied across knowledge domains, with relatively lower accuracy observed in internal medicine and emergency and critical care questions compared with other examination categories. Taken together, the findings suggest that current LLMs perform relatively well on structured nursing knowledge assessments, although performance remains variable across different task types and clinical domains.

### Error Patterns and Clinical Reasoning Challenges

Review of incorrect responses suggested that some challenges may have been associated with scenarios involving oncology-specific nursing knowledge, complex clinical reasoning, emergency management, and threshold-based clinical decisions. Because these observations were derived from qualitative review rather than a predefined error classification framework, they should be interpreted cautiously. Nevertheless, they may provide useful insights into clinical situations that remain challenging for current LLMs [21]. Tofeeq et al [9] reported that LLMs generally achieve high accuracy in basic medical question–answering tasks but their performance is uneven across different task dimensions, particularly in complex reasoning and highly specialized domains. These findings suggest that the limitations of LLMs in oncology nursing may not be related only to knowledge coverage. Rather, they may also reflect the difficulty of applying general knowledge to context-sensitive clinical situations. Oncology nursing requires not only factual knowledge but also the integration of dynamic clinical information, including symptom progression, treatment stage, and patient-specific conditions. Similar limitations have been reported in other oncology-related applications of LLMs, where performance may decline when tasks require interpretation and management of complex treatment-related information [22]. Clinical scenarios involving urgent decision-making or interpretation of clinical thresholds may be particularly difficult for current LLMs. Such situations require accurate interpretation of patient information and timely prioritization of actions. Previous systematic reviews have highlighted limitations related to knowledge coverage, contextual adaptability, and safety [23]. In the present study, difficulties appeared more evident in scenarios involving case analysis, integrative reasoning, numerical thresholds, and contraindication judgments. Some model-generated responses included clinically inappropriate recommendations in these scenarios even when they appeared logically coherent. This limitation has also been reported in previous evaluations of LLMs in health care settings [24,25]. One possible explanation is that LLMs generate responses based on probabilistic language patterns and may therefore produce recommendations that appear plausible but lack the precision required for clinical decision-making. This may partly explain the observed difficulties in clinically complex scenarios despite generally acceptable performance on structured knowledge-based tasks [1].

These observations also point to the distinctive demands of nursing decision-making. Unlike diagnosis-oriented tasks, nursing practice requires continuous patient monitoring, risk prioritization, and individualized interventions. Therefore, evaluation of LLMs in nursing should prioritize context sensitivity, temporal dynamics, and risk stratification. These processes are highly context dependent and require integration of multiple sources of clinical information. In oncology nursing, this complexity is further amplified by rapidly evolving oncologic situations and treatment-related toxicity profiles [2]. As a result, the limitations of LLMs extend beyond incomplete knowledge coverage to include difficulties in translating knowledge into actionable nursing decisions. This aligns with previous research indicating that LLMs may produce outputs that are linguistically coherent but not always clinically appropriate [24]. From a practical perspective, current LLMs may be better suited to supportive functions such as information retrieval and patient education than to independent clinical decision-making in oncology nursing settings.

### Differences Across LLMs

In terms of model comparison, DeepSeek achieved the highest score in the case-based evaluation, whereas the remaining models showed comparatively lower performance. This pattern differs from some findings reported in English-language settings. Differences in language environment, training data distribution, and domain-specific corpus coverage may partly explain the variation in model performance [6]. Consistent with this interpretation, Lai et al [26] reported that LLM performance is highly dependent on the language structure and domain coverage of the training corpus.

Models with stronger Chinese-language and domain-specific training may therefore have been better able to capture local clinical terminology and practice patterns in Chinese nursing scenarios, which is broadly consistent with findings reported in studies of Chinese medical LLMs [27]. An additional observation was the discrepancy in ChatGPT’s performance across the 2 evaluation tasks. Although ChatGPT achieved the second-highest accuracy in the standardized examination assessment, its performance in the case-based correctness, clarity, and conciseness evaluation was comparatively lower. This observation suggests that performance on structured examination questions may not necessarily translate into stronger performance in case-based nursing scenarios. Standardized examination questions tend to emphasize recognition of predefined answers and structured knowledge application. In contrast, case-based questions require integration of contextual information, prioritization of nursing problems, and application of knowledge within dynamic clinical situations. These tasks place different cognitive demands on LLMs. Therefore, the observed discrepancy may reflect differences in task characteristics rather than differences in knowledge representation alone. These results indicate that examination performance alone may not adequately reflect model performance in clinically contextualized nursing scenarios [9]. This discrepancy highlights the value of including clinically contextualized case scenarios when evaluating LLM performance in nursing. Although DeepSeek scored significantly higher than ChatGPT, no significant differences were observed among the remaining pairwise comparisons after adjustment for multiple testing. The relatively small number of case-based questions, together with correction for multiple comparisons, may have limited the ability to detect smaller performance differences between models. Therefore, the absence of statistical significance should not be interpreted as evidence of equivalent performance. Larger and more diverse question sets may help determine whether additional performance differences exist among models. Consistent with previous studies, the present findings suggest that current LLMs retain important limitations when applied to complex clinical reasoning tasks [9]. Moreover, none of the evaluated models achieved a consistently error-free level of performance in the present study. Differences in the number of sessions required to obtain complete responses were also observed across the evaluated platforms. Because all models were accessed through publicly available web-based interfaces, these observations may reflect characteristics of the web-based platforms in addition to the models themselves. However, the present study was not designed to evaluate platform-level factors, and the reasons for these differences remain unclear. Accordingly, the number of sessions required to obtain complete responses should not be interpreted as a direct measure of intrinsic model capability.

### Interpretation of Correctness, Clarity, and Conciseness Scores and Rating Reliability

Interpretation of the correctness, clarity, and conciseness evaluation results should take the prompting strategy into account. All models were instructed to provide concise final answers without explanation, and the same prompt was applied across both case-based and examination questions. Gilson et al [18] adopted a standardized prompting strategy when evaluating LLM performance. Similar evaluation approaches have been adopted in studies assessing LLM performance in health care settings, where evaluation has primarily focused on generated outputs [9]. Therefore, the clarity and conciseness dimensions should be interpreted as indicators of final response quality under a standardized prompting condition rather than as direct assessments of detailed clinical reasoning, reasoning transparency, or broader nursing communication ability [28]. Future evaluations using case-based nursing scenarios may benefit from prompts designed to elicit step-by-step reasoning when reasoning transparency is the outcome of interest. A separate issue concerns the reliability of the subjective evaluation process. Using the Landis and Koch interpretation scale for agreement coefficients, the weighted κ values for correctness, clarity, and conciseness fell within the moderate range. The ICC for the total correctness, clarity, and conciseness score also indicated moderate reliability. Although these results suggest an acceptable level of agreement for an exploratory evaluation, some variability remained between evaluators. This may partly reflect the subjective nature of assessing response quality, particularly for dimensions such as clarity and conciseness, which are inherently more open to interpretation than factual correctness. Hallgren [29] noted that variability between raters is common when subjective judgment is involved even when standardized evaluation criteria are used. Therefore, the subjective evaluation results should be interpreted with appropriate caution. Given the subjective nature of some assessment dimensions, the use of more detailed scoring rubrics and structured rater calibration procedures may help improve consistency in future evaluations [29].

### Implications for Oncology Nursing Practice

The findings suggest that LLMs may have practical value in oncology nursing when used for structured information processing, educational support, and routine communication tasks. Song et al [23] reported that LLMs can achieve relatively high accuracy in standardized medical examinations, particularly in structured question-answering tasks. Will et al [30] further showed that LLMs may improve the readability and comprehension of complex medical information. These findings support the potential use of LLMs as supplementary tools in nursing education and patient education. In oncology nursing, such tools may help organize complex information, summarize standardized care protocols, and prepare patient-facing educational materials. The integration of LLMs into oncology nursing practice raises important considerations regarding patient safety, ethical responsibility, and accountability. Because nursing decisions may directly affect patient outcomes, clear frameworks for risk management and responsibility attribution will be necessary before broader clinical implementation can be considered [31]. In clinical practice, LLMs may be most appropriately integrated into specific components of the nursing workflow rather than the entire clinical decision-making process. They may assist with organizing symptom-related information and summarizing risk factors during patient assessment, supporting structured documentation of treatment-related adverse events during treatment, and generating discharge instructions or self-care guidance for patient education. These functions primarily support information organization and communication rather than direct patient care decisions. With appropriate governance frameworks and professional oversight, LLMs may contribute to more efficient clinical information management and documentation processes in oncology nursing settings. However, their application should remain clearly bounded within a supportive rather than autonomous role. Particular caution may be required in complex oncology nursing situations, such as toxicity monitoring, infection risk assessment, and dynamic symptom management during cancer treatment. These scenarios often require integration of patient-specific information and clinical context, which may present challenges for current LLMs. Future studies should focus on domain-specific fine-tuning using oncology nursing guidelines and clinical pathways together with validation in real-world clinical environments. Such work may help clarify the role of LLMs in oncology nursing practice and support their safe and effective implementation.

### Limitations

This study has several limitations. Although all eligible case-based questions from the selected training manual were included, the overall number of questions remained limited. This may have reduced statistical power for pairwise comparisons and made it harder to detect smaller performance differences among models, particularly after adjustment for multiple testing. The evaluation was based on standardized test items, which ensured comparability across models but may not fully reflect performance in real-world clinical settings characterized by multimorbidity, dynamic disease progression, and incomplete information. Although oncology-related content was included, highly complex scenarios specific to oncology nursing, such as cross-system coordination and long-term care decision-making, were underrepresented. The examination assessment relied on a commercially published preparation resource rather than official examinations. Although the resource was developed for preparation for the Chinese Nursing (Intermediate) Qualification Examination, its representativeness of current examination standards cannot be fully guaranteed. Potential overlap between some evaluation items and materials that may have been accessible during model training also cannot be excluded. Because the training data of the evaluated LLMs are not publicly available, the extent of possible memorization could not be assessed directly. Several methodological factors should also be considered. Differences in training data, reasoning strategies, and sensitivity to question phrasing may also have influenced the results. A uniform prompt was used to ensure comparability across models, but this approach may have underestimated performance under optimized prompting conditions. Each model was queried only once for each question, and repeated sampling was not performed. Response variability across repeated runs was therefore not assessed. In addition, specific model version identifiers could not be retrospectively verified for all platforms. Updates to publicly available models during the data collection period cannot be completely ruled out, and their potential influence on model performance could not be assessed directly. This study did not include a human comparator group, which limits direct comparison between model performance and clinical practice. Regional differences in health care systems, clinical guidelines, and cultural contexts were also not considered and may affect the generalizability of the findings.

### Conclusions

All evaluated LLMs achieved passing scores on the standardized examination assessment, but performance varied across case-based oncology nursing scenarios. DeepSeek achieved the highest performance in the case-based evaluation, whereas performance on examination questions did not consistently correspond to performance in clinically contextualized assessments. Limitations were most evident in scenarios requiring clinical reasoning, contextual interpretation, and risk-sensitive decision-making. These findings suggest that performance on standardized examination questions alone may not adequately reflect the ability of LLMs to support nursing practice in real-world clinical settings. These findings indicate that examination-style testing may be complemented by clinically contextualized case scenarios when evaluating LLMs in nursing. From a practical perspective, current LLMs may be better suited to tasks such as information retrieval, knowledge organization, and patient education than to independent clinical decision-making. Further progress in this area will require domain-specific development and rigorous clinical validation. Appropriate governance frameworks will also be essential for the safe integration of LLMs into oncology nursing practice.

## Abbreviations

ICC: intraclass correlation coefficient
LLM: large language model
STROBE: Strengthening the Reporting of Observational Studies in Epidemiology
